# Seeking global agreement on controlled donation after circulatory determination of death: methodology and definitions of the Bucharest international European Society for Organ Transplantation (ESOT) consensus

**DOI:** 10.3389/ti.2026.16280

**Published:** 2026-05-28

**Authors:** Dominique E. Martin, Umberto Cillo, Marius Berman, Alexandra K. Glazier, Eduardo Miñambres, Helen Opdam, Alicia Pérez-Blanco, Francesco Procaccio, Marion Siebelink, Matthew J. Weiss, Beatriz Domínguez-Gil, Gabriel C. Oniscu

**Affiliations:** 1 Faculty of Health, Deakin University, Geelong, VIC, Australia; 2 Unità di Chirurgia Epatobiliare e Trapianto Epatico, Università degli Studi di Padova, Padova, Italy; 3 Royal Papworth Hospital NHS Foundation Trust, Cambridge, United Kingdom; 4 New England Donor Services, Waltham, MA, United States; 5 Brown University School of Public Health, Providence, RI, United States; 6 Transplant Coordination Unit and Service of Intensive Care, University Hospital Marqués de Valdecilla-IDIVAL, Santander, Spain; 7 Universidad de Cantabria Facultad de Medicina, Santander, Spain; 8 Austin Health, Heidelberg, VIC, Australia; 9 Australian Organ and Tissue Authority, Canberra, VIC, Australia; 10 Organizacion Nacional de Trasplantes, Madrid, Spain; 11 Fondazione Trapianti Onlus, Milan, Italy; 12 Universitair Medisch Centrum Groningen, UMCG Transplantatiecentrum, Groningen, Netherlands; 13 Transplant Quebec, Montreal, QC, Canada; 14 Division of Transplantation Surgery, CLINTEC, Karolinska Institutet, Stockholm, Sweden

**Keywords:** consensus, controlled donation after circulatory determination of death (cDCDD), ethics, organ donation, transplantation

## Abstract

Controlled donation after circulatory determination of death (cDCDD) is now a major contributor to transplant activity worldwide. However, its expansion has been complicated by inconsistent terminology, diverse clinical protocols, and ethical uncertainties. To address these challenges, the European Society for Organ Transplantation (ESOT) convened a global consensus forum involving multidisciplinary experts from various geographic regions. Four steering committees were established to address adult and pediatric donation pathways, normothermic regional perfusion, and the determination of death. Using a structured methodology, expert panels participated in two waves of surveys, complemented by an in-person consensus meeting to develop recommendations on key clinical and ethical aspects of cDCDD. This article presents the project methodology and consensus results regarding standardized terminology and definitions that are essential for successfully harmonizing international practice. Consensus was achieved on fundamental terms, including the recommended nomenclature of “donation after circulatory determination of death,” definitions of clinical categories such as possible and potential donors, and key time points in donation protocols. The establishment of shared terminology provides a foundation for comparing outcomes across programs, facilitating international research collaboration, and supporting evidence-based improvements in clinical practice. This consensus represents an important step toward global convergence of donation practices while maintaining core ethical values and supporting continued technological and societal progress in the field.

## Introduction

The sustained growth of controlled donation after circulatory determination of death (cDCDD) has been the main contributor to the recent increase in transplant activity [1]. In 2024, cDCDD comprised 27% of global deceased organ donation activity [1]. cDCDD, in which organs are removed after death occurs following the planned withdrawal of life-sustaining measures (WLSM), has been fueled by major technological developments in organ perfusion and preservation, along with the cultural evolution of end-of-life care [2, 3]. However, the development of cDCDD appears to be at an inflection point as a consequence of mixed clinical results, diverse and sometimes inconsistent protocols, ethical and legal controversies, and uncertainties regarding various practices [4, 5]. Consequently, a global consensus forum was conceived by the European Society for Organ Transplantation (ESOT) with the overarching objective of defining good practices and standards in cDCDD with respect to adult and pediatric donor management, use of normothermic regional perfusion (NRP), and the determination of death in the cDCDD context. The project aimed to provide actionable recommendations to address key clinical and ethical uncertainties or controversies, thereby optimizing opportunities for the successful recovery and transplantation of organs from cDCDD donors in countries at various stages of cDCDD program development.

Deceased donation programs and practices are shaped by local legal and policy frameworks and healthcare system design and resources, in addition to professional and societal cultures and norms with regard to end-of-life care and decision-making [6]. The international diversity of cDCDD programs consequently complicates efforts to systematically review evidence of good practices and achieve professional consensus regarding ethical standards of care; these challenges are exacerbated by the use of heterogeneous terminology and definitions. Accordingly, establishing consensus on key terms and definitions emerged as an important secondary objective of the project.

In this study, we outline the methodology of the project and briefly present the results pertaining to the definitions of common terms of interest, which are collated from the work of the four steering committees (SCs) dedicated to adult and pediatric cDCDD pathways, the NRP, and the determination of death. The remaining results are presented in the reports of the four SCs [7–10]. The key recommended definition of ante-mortem interventions for donation and its significance are discussed in depth in the context of the adult cDCDD pathway [7]; definitions of the unifying concept of death (UCD) and the “dead donor rule” are similarly explored [8]. Here, we also discuss the limitations of the project and explore future goals and developments, along with unmet needs, that are essential for defining clinical and research priorities. This consensus conference is an important step toward the global convergence of cDCDD practices; it provides a foundation of clinical and ethical norms that support technological and societal progress while upholding the longstanding core values of donation and transplantation [11].

## Methods

**FIGURE 1:**
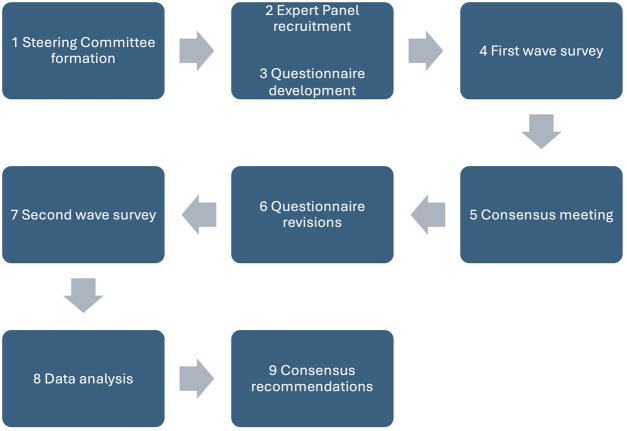
Summary of the key steps in the Consensus Project.

Figure 1 provides the overall schema of the Consensus project. Four SCs were assembled to lead the work on the adult and pediatric cDCDD pathways, the NRP, and the UCD. The workstream coordinators appointed six to eight members to each committee in collaboration with the project co-chairs. SC members were selected for their expertise and relevant experience, ensuring professional diversity and representation from various geographic regions.

### Expert panel recruitment

Each committee nominated panelists who were invited via email to participate in the first-wave survey conducted by Adelphi Targis, a company that uses Delphi methodology to support clients in the development of guidelines [12].

Purposive sampling was used to ensure breadth in disciplinary expertise (e.g., intensive care, ethics, donor coordination, transplant surgery) and geographical representation. The inclusion criteria for panelists were as follows:

- Expertise and professional experience of relevance to the topic of the subgroup, as assessed by evidence of scientific publications and/or professional appointments.
- Sufficient English proficiency to complete the survey questionnaire.

All committee members except the coordinators were also included as panelists. All panelists who completed the first questionnaire were invited to complete the second-wave survey. Adherence was between 95% and 100% (see Table 1).

**TABLE 1:** Number of panelists who were invited and completed the survey rounds for each group.

Item	Adult DCD pathway	Pediatric DCD pathway	Unifying concept of death	Normothermic regional perfusion
No. of SC members, including two coordinators	9	8	8	10
No. of panelists invited	43	35	48	31
No. of panelists who completed the first-wave questionnaire	37	30	42	30
No. of panelists who also completed the second-wave questionnaire	35	30	40	30

Some individuals were invited to participate in two panels. Surgeons were excluded from participation in the UCD panel to minimize perceived or actual conflicts of interest in developing recommendations for the determination of death in the context of cDCDD. The demographics for each panel are detailed in the reports of each group [7–10].

### Questionnaire development

Each SC developed a questionnaire that addressed a range of topics identified as priorities via a scoping review of the literature and group discussions. The wording of the items was refined following committee discussions. The questionnaires were further revised to align relevant items between questionnaires for groups with subtopics of mutual interest, in particular those relating to the definition of terminology. The co-Chairs reviewed all questionnaires before finalization to ensure consistency of terminology.

Each questionnaire comprised an initial common section with basic demographic items. Statements for which consensus was sought were organized thematically and presented with a nine-point Likert scale eliciting levels of (dis)agreement (1-9, strongly disagree to strongly agree). An option was provided to the participants to indicate if a question was not relevant to their area of expertise. The final section of each questionnaire provided space for free-text comments. In the UCD questionnaire, only respondents with relevant clinical expertise were assigned questions relating to the clinical aspects of the determination of death in the adult and pediatric contexts.

### Consensus meeting and questionnaire revision

The results of the first-round survey were reviewed and discussed by members of each SC, panelists, and attendees at the ESOT DCD Consensus meeting, held in Bucharest, Romania, on 12 October 2024. This in-person meeting was essential for receiving comments from the professional community and developing the second survey, which focused on statements that did not reach consensus in the first wave. The questions in the second survey were finalized by the coordinators and co-chairs following the meeting.

When developing the questionnaire for the second wave survey, items that had achieved consensus or that were deemed redundant were removed. Redundant items were those presented as alternative statements for which a clear preference was indicated in support of a specific formulation. Some remaining items were revised in view of the discussions in Bucharest to improve clarity or specificity regarding minimum standards or to consolidate foundational principles; some items were added to address key recommendations introduced by experts during the meeting. The wording and structure of some questions and statements were revised to facilitate the expression of preferred options or clear recommendations. For the UCD questionnaire, the clinical pediatric section was removed due to the insufficient number of pediatric respondents in the first wave [8].

Questions regarding definitions and preferred terminology were included in the first section of the second-wave questionnaires. Due to the different focus of the four groups, not all items regarding definitions were presented to each panel.

### Scope

Of note, while donation after medically assisted death (euthanasia) is a form of cDCDD and was included in the definition of a potential donor agreed upon during this project (see below), the focus of the project was cDCDD that occurred following the planned WLSM. Nevertheless, the majority of the recommendations produced by the four working groups may be considered applicable to donations that occur following assistance in dying.

### Data collection

The survey was hosted online by Adelphi Targis. Panelists were given at least 1 month to complete the questionnaire during each round. Adelphi Targis sent follow-up emails to prompt completion of the questionnaires in both rounds.

### Data analysis

The responses were analyzed by Adelphi Targis using descriptive statistics. “Disagreement” was assigned to ratings 1–3, “neither agree nor disagree” to ratings 4-6, and “agreement” to ratings 7–9. Responses from those who indicated that they lacked relevant expertise were removed from the denominator when evaluating consensus on specific items. Statements that reached 75% agreement or disagreement were deemed to have achieved consensus.

When analyzing the results of the UCD questionnaire from the first wave, responses from pediatric experts for the section focused on clinical considerations in the determination of death were excluded from analysis due to the small number of respondents (n = 4).

When analyzing the results of the second-wave survey, neutral responses were excluded when assessing consensus on a limited number of new or revised questions that were designed to elicit a preferred response. Since selecting a specific statement from a set of alternatives was not possible due to the format of the survey platform, the respondents were advised to indicate their preferred option or the statement they considered “best” by ensuring their responses to these statements were internally consistent. For example, if a respondent strongly agreed with a first statement indicating that ‘the best definition of the practice is A”, then it was expected that they would strongly disagree with a second statement indicating that ‘the best definition of the practice is B”. Nevertheless, approximately 20% of respondents neither agreed nor disagreed with several such statements. When analyzing responses to this type of question, neutral responses were excluded when assessing consensus, as they did not indicate a clear directional preference.

## Results

Consensus was achieved for all proposed terms and definitions, resulting in the recommended terminology presented in Tables 2, 3. In addition, there was consensus that the best term for donation, which occurs following the withdrawal of life-sustaining measures (WLSM) and circulatory arrest, is [controlled] “donation after circulatory determination of death.”

**TABLE 2:** Consensus recommendations for the definition of key terms in cDCDD.

Term	Definition in the context of cDCDD
Substitute decision-maker (SDM)	The person(s) with the legal authority to make end-of-life and/or donation decisions on behalf of the patient or potential donor who lacks [decision-making] capacity
Family (of a possible or actual donor)	Those closest to the person in knowledge, care, and affection. This includes the immediate biological family; the family of acquisition (related by marriage/contract); and the family of choice and friends (not related biologically or by marriage/contract)
Possible donor	A person approaching the end of life, who may be a potential donor but whose suitability for cDCDD has not yet been formally evaluated
Potential donor	A person considered medically suitable to donate, for whom WLSM or medically assisted death (euthanasia) is planned, with circulatory arrest expected to occur within a time frame that will enable organ recovery
Medically suitable for donation	A person with no clinical contraindications for donation who is expected to have one or more organs suitable for transplantation
Ante-mortem intervention for donation	Any clinical procedure or test that is performed before death for the purpose of organ or tissue donation and transplantation that would not occur in the absence of consideration of donation
Functional perfusion of an organ or tissue	The passage of blood or other fluids through blood vessels that results in the exchange of oxygen, solutes, and/or nutrients to the cells of the organ or tissue and the removal of cellular waste

**TABLE 3:** Definition of key time points in the context of cDCDD protocols.

Term	Definition
Agonal phase	The period between the WLSM and circulatory arrest
Withdrawal of life-sustaining measures (WLSM)	The cessation of all cardiorespiratory support, including mechanical ventilation, vasoactive agents, and/or any mechanical circulatory devices
Circulatory arrest	The absence of pulsatile blood flow following WLSM.
Onset of circulatory arrest	When there is a loss of pulsatile blood flow (mechanical asystole) following WLSM.
Acirculatory phase	The period between circulatory arrest and the start of cold perfusion or, when used, normothermic regional perfusion (NRP)
Total warm ischemia time	Begins when life-sustaining measures are withdrawn and ends with the start of cold perfusion or, when used, NRP.
Functional warm ischemia time (FWIT)	The period between the onset of sustained organ hypoperfusion (as specified by local or national guidelines) and the start of cold perfusion or, when used, NRP.
	Consensus recommendations: Systolic blood pressure and/or oxygen saturation measures of hypoperfusion that define the start of FWIT should be standardized internationally National or local protocols should establish organ-specific standards that define the maximum duration of FWIT within which organs should be successfully recovered and transplanted
Cold ischemia time	The period between the commencement of cold perfusion of an organ and the restoration of blood supply to the organ
No touch period	Mandated minimum period of time following circulatory arrest before death is declared and organ retrieval can begin
Stand-down time	The time when an attempt at DCDD is aborted because death has not occurred within a timeframe that enables organ eligibility for transplantation, as defined by local protocols

## Discussion

The rapid expansion of cDCDD and the use of machine perfusion technology for organ recovery have occurred with significant heterogeneity in terminology, leading to challenges in comparing international practices. As such, one of the key aims of this consensus project was to propose standardized terminology and definitions to facilitate further, more precise comparisons, cooperation, and the development of cDCDD programs globally.

Semantic consensus regarding the best term for donation that occurs following WLSM and circulatory arrest provides a strong foundation for further legal and policy work in defining death and harmonizing cDCDD protocols. Despite the longstanding recognition that the term ‘donation after circulatory determination of death’ is “most precise”, previous consensus work in 2013 concluded that the simpler phrase “donation after circulatory death” was preferable [13]. While this may appear to be an efficient abbreviation in comparison to DCDD, omitting “determination of” has problematic implications. It contributes to the perception that there are two types of death, i.e., that an individual may have suffered “circulatory death”, but not “brain death” or *vice versa*. In contrast, by specifying donation after the circulatory *determination* of death, the recommended term clearly links the donation pathway to a particular process of death determination rather than to clinical conditions at the time of donation. In addition, the term “donation after cardiac death” remains widespread, despite its problematic implications. For example, when a heart is recovered via the cDCDD pathway and used in transplantation, this may be perceived as incompatible with donation after “cardiac death” because the heart is evidently physiologically functional after the declaration of death. Although consensus was not sought on terminology pertaining to donation after “brain death”, it is reasonable to infer that the term “donation after neurological determination of death” or DNDD should be adopted in conjunction with DCDD.

The consensus achieved on definitions of key clinical categories or time points in donation protocols, such as ‘possible donor’ or ‘functional warm ischemia time’ (FWIT), likely reflects an avoidance of definitions that were tied to specific clinical criteria. While panelists agreed on the descriptive definition of FWIT, for example, consensus was not reached on the strict clinical thresholds that should define the start of FWIT. These measures may evolve over time with increased understanding of the potential impact–or lack thereof - of specific thresholds on transplant outcomes, especially in the context of perfusion technologies such as NRP. Furthermore, clinical disagreement on the appropriate standards can be problematic if international recommendations for clinical protocols are grounded in specific definitions for which consensus may be lacking. A previous consensus definition of FWIT, for example, described it as “the time from when the systolic blood pressure drops below 50 mmHg (irrespective of oxygen saturation) for at least 2 min … ” [SIC] following WLSM [13]. However, studies have indicated that oxygen saturation may be a key predictor of graft function for some organs [14], and no clear consensus on the appropriate clinical measures of FWIT is evident from the literature [15]. As such, panelists recommended standardization of hypoperfusion measures that define the start of FWIT, with the specific thresholds being at the latitude of local or national guidelines.

The distinction between a possible and a potential donor in the recommended definitions provides scope to harmonize the evaluation of the capacity for cDCDD donation internationally and could be helpful in improving the identification of individuals who could become donors. A “possible” donor is notably defined as “a person approaching the end of life, who may be a potential donor but whose suitability for cDCDD has not been formally evaluated.” In contrast, in the context of cDCDD, a “potential donor” describes a “person considered medically suitable to donate, for whom WLSM or medically assisted death (euthanasia) is planned, and circulatory arrest is expected to occur within a time frame that will enable organ recovery.” These definitions align with previous definitions of possible and potential donors, such as those by Dominguez-Gil et al. [16] However, the new recommended definitions allow for consideration of donation opportunities by individuals previously excluded by some definitions, such as those who lack a devastating brain injury or circulatory failure, or those who might otherwise be deemed medically unsuitable to donate prior to a more formal evaluation of suitability.

The impact of heterogeneity in definitions and terminology was evident in this project through the disagreements observed, especially in first-wave surveys. When exploring disagreement, for example, about when or if "possible" donors should be referred to donation professionals, it became clear that some panelists held ethical concerns about potential conflicts of interest that might arise when referrals were made. This was because the term "referral" had specific practical implications in some settings which meant that early "referral" might create the perception of a conflict of interest in decision-making about end of life care. Seemingly innocuous terms, such as “referral” or “notification,” which are prevalent in the literature when describing donation protocols, were found to have specific practical or legal implications in certain settings, which could raise concerns not apparent in different jurisdictions or donation programs. Global implementation of standard terminology is thus essential for harmonizing clinical protocols and data, facilitating research, and informing best clinical practices and ethical standards. The additional importance of establishing global clinical standards was evident in the recommendations of the NRP and UCD groups [8, 9]; however, this consensus project highlights the difficulty of achieving this when even minor differences in clinical practices or healthcare systems can substantially influence clinical outcomes and ethical considerations, such that substantive disagreement may be evident among expert peers working in different settings.

To support more effective cooperation and collaboration in the development of cDCDD programs and research activities internationally, consensus and adoption of a shared language to describe protocols and measure outcomes, in addition to consensus on principles that should underpin practice regardless of contextual diversity, are essential. The results of this consensus project demonstrate the potential for widespread agreement on principle-based norms in cDCDD, the professional community’s belief in the importance of establishing global clinical standards in cDCDD, and the challenges that must be considered when seeking international consensus in a rapidly evolving field of clinical practice that is heavily influenced by clinical, legal, and sociocultural elements in every country.

### Limitations

The use of the Delphi methodology in this study helped to elicit expert opinions on a range of topics, many of which lack a strong empirical evidence base and some of which are controversial or multidisciplinary in nature. The structured approach to questionnaire development and the implementation of the two survey waves, coupled with the rich discussions during the Bucharest meeting, supported open consideration of diverse views and experiences. This aligned well with the broad scope of the project, which aimed to address complex cDCDD aspects from an international perspective.

However, the wide diversity of experience and expertise among panelists was a significant limitation, particularly in the context of questionnaire topics focused on clinical aspects of cDCDD or the determination of death. Despite robust discussions during the Bucharest meeting and among steering committee members, consensus was not achieved on several points. Furthermore, the absence of consensus was not always explicable, suggesting that efforts to improve the clarity and specificity of the questionnaire items were sometimes unsuccessful or failed to account for the impact of unknown contextual factors.

Where consensus was achieved, including the definitions of terms reported in this article, it is important to note the limitations of the panelist population. The majority of panelists were nominated by peers, and thus, the sample of views represented may be biased. Nevertheless, the consensus recommendations from all four groups are largely consistent with related recommendations in peer-reviewed literature grounded in empirical evidence and expert consensus work on more focused topics within the scope of cDCDD.

## Conclusion

cDCDD continues to expand globally and could become the primary pathway to organ donation in many countries, supported by technological developments in *in situ* and *ex situ* organ perfusion. The field of donation and transplantation is rapidly evolving, and this timely consensus project enabled stimulating and productive conversations on key topics and unresolved ethical concerns in cDCDD. While consensus was achieved in some areas, continued dialogue is needed to explore local and regional variations in practice, to support international research collaborations that will improve clinical practice, to benchmark activities and outcomes, and to enrich collective learning and knowledge.

The ESOT recently adopted the concept of a Society-driven Consensus Platform to facilitate the dynamic scientific developments and their implications for clinical practice and to promote clinician alignment. This structure will provide a valuable tool to enable further international work on cDCDD, especially when coupled with meetings dedicated to open dialogue and transformative collaboration at the global level.

## Data Availability

The raw data supporting the conclusions of this article will be made available by the authors, without undue reservation.
